# The Catacombs of Paris

**Published:** 1881-06

**Authors:** 


### THE CATACOMBS OF PARIS.

The vast catacombs by which a large portion of the city of Paris are undermined were only known by popular tradition until the year 1774, when some alarming accidents aroused the attention of the Government. The old quarries were then surveyed and plans of them taken, and the result was the frightful discovery that the churches, palaces and most of the southern part of Paris was undermined, and in great danger of sinking into the pit below them. A special commission was appointed, and on the very day it met a house in one of the streets sunk ninety-one feet below the level of its court-yard. The pillars which had been left by the quarrymen, in their blind operations, without any regularity, were in many places too weak for the enormous weight above, and in most places had themselves been undermined, or perhaps originally stood upon ground which had previously been hollowed. The aqueduct of Arcueil passed over this treacherous ground; it had already suffered some shocks, and, if the quarries had continued to be neglected, an accident must, sooner or later, have happened to this water-course, which would have cut off its supply from the fountains of Paris, and have filled the excavations with water. Repairs were forthwith commenced and promptly completed, and a portion of the old quarries was devoted to receive the bones of the dead. This took place in April, 1786; the remains of the dead were removed at night in funeral cars, covered with a pall, and followed by priests chanting the service of the dead. When they reached the catacombs the bones were shot down a well, and the rattling and echoing which they made in their fall were as impressive as any sound ever heard by human ears. Thus the limestone quarries that had supplied the materials for building the superb monuments, palaces and houses of Paris became huge charnel-houses, which they now remain. Calculations differ as to the number of bones collected in the catacombs, but it is certain that they contain the remains of at least 3,000,000 of human beings.—*Harper's Young People.*

### THE BAGDAD DATE MARK.

Bagdad, says one of our medical exchanges, is noted for a curious and mysterious malady, which affects everybody in the city, whether he be citizen or stranger. It is a sore called a "date mark," because after it has healed it leaves an indelible mark about the size and shape of a date. It generally makes its appearance upon the face, lasts a year and then disappears. The cheek of nearly every man and woman in Bagdad shows the inevitable mark. Sometimes it settles upon the nose, and then the disfigurement is great; sometimes on the eyelid, when blindness is the result. Strangers are attacked even after a brief residence; but, fortunately, if they are adults, the sore is more apt to come on the arm. In every case the attack runs its course for one year. No treatment, no ointment, nor medicine, it is said, has the slightest effect upon it. Once the sore appearing, the sufferer knows what to expect, and may as well resign himself to his fate. The Arabs say that every one who goes to Bagdad must get the "date mark"; or, if he does not get it while in the city, he will be followed by it—have it sooner or later, he must. Dr. Thom, of the American mission, states that he has examined the ulcer microscopically, and found it to be composed of a fungoid growth, but nothing that he has ever tried has proved remedial.

